# Epigenetic Alterations as Vital Aspects of Bortezomib Molecular Action

**DOI:** 10.3390/cancers16010084

**Published:** 2023-12-23

**Authors:** Piotr Kulig, Karolina Łuczkowska, Estera Bakinowska, Bartłomiej Baumert, Bogusław Machaliński

**Affiliations:** 1 Department of General Pathology, Pomeranian Medical University, 70-111 Szczecin, Poland; piotrkulig@interia.eu (P.K.); karolina.luczkowska@pum.edu.pl (K.Ł.); esterabakinowska@gmail.com (E.B.); 2 Department of Hematology and Transplantology, Pomeranian Medical University, 71-252 Szczecin, Poland

**Keywords:** bortezomib, proteasome inhibitor, drug resistance, epigenetic, methylation, non-coding RNA

## Abstract

**Simple Summary:**

The implantation of proteasome inhibitors was a milestone in the treatment of multiple myeloma (MM). One such first-in-class molecule was bortezomib (BTZ). Its cytotoxic effects are exerted through proteasome inhibition and the subsequent accumulation of misfolded or otherwise defective proteins. In addition to its main mechanisms of action, BTZ elicits various epigenetic alterations within target cells which are part of its mechanism of action. Importantly, epigenetic changes also participate in mediating resistance to BTZ. Some epigenetic agents such as azacitidine act synergically with BTZ or have the potential to restore sensitivity to the drug in resistant malignant cells. In this paper, we reviewed the epigenetic aspects of BTZ molecular action with a particular emphasis on drug resistance mechanisms and potential clinical implications.

**Abstract:**

Bortezomib (BTZ) is widely implemented in the treatment of multiple myeloma (MM). Its main mechanism of action is very well established. BTZ selectively and reversibly inhibits the 26S proteasome. More precisely, it interacts with the chymotryptic site of the 20S proteasome and therefore inhibits the degradation of proteins. This results in the intracellular accumulation of misfolded or otherwise defective proteins leading to growth inhibition and apoptosis. As well as interfering with the ubiquitin–proteasome complex, BTZ elicits various epigenetic alterations which contribute to its cytotoxic effects as well as to the development of BTZ resistance. In this review, we summarized the epigenetic alterations elicited by BTZ. We focused on modifications contributing to the mechanism of action, those mediating drug-resistance development, and epigenetic changes promoting the occurrence of peripheral neuropathy. In addition, there are therapeutic strategies which are specifically designed to target epigenetic changes. Herein, we also reviewed epigenetic agents which might enhance BTZ-related cytotoxicity or restore the sensitivity to BTZ of resistant clones. Finally, we highlighted putative future perspectives regarding the role of targeting epigenetic changes in patients exposed to BTZ.

## 1. Introduction

**Figure 1:**
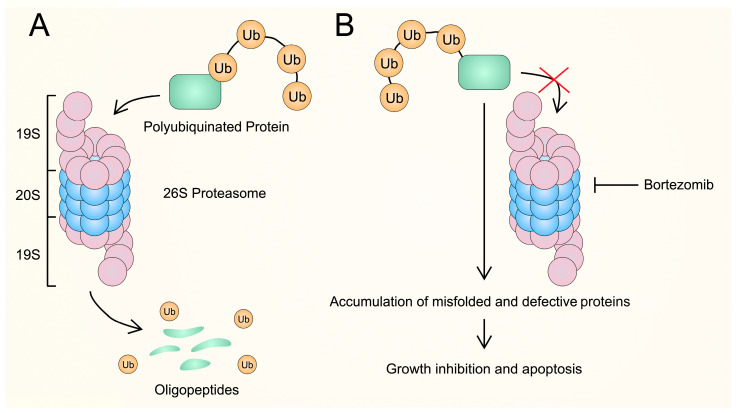
Proteasome activity without and with the presence of BTZ. (**A**) Proteasome degrades polyubiquitinated proteins into oligopeptides. (**B**) BTZ inhibits the 26S proteasome, leading to an intracellular accumulation of misfolded or otherwise defective proteins, and subsequent growth inhibition and apoptosis.

Proteins are crucial components for maintaining intracellular homeostasis. Their role is multifaceted, as they participate in numerous, if not all, biological processes in cells. Due to the complexity of the interactions in which proteins are involved, it is extremely important to regulate their metabolism, in particular, their synthesis and turnover. Protein synthesis is closely related to the regulation of translation and the bioavailability of various RNA molecules [1], whereas protein degradation and turnover are controlled through the lysosomes or the ubiquitin–proteosome system (UPS). The lysosome-based degradation pathways involve the degradation of cytoplasmic proteins and damaged organelles. This process occurs through autophagy, known as the autophagy-lysosomal pathway, and the degradation of extracellular proteins through endocytosis, referred to as the endosome-lysosomal pathway [2]. A particular pathway is the phagocytosis-lysosome pathway, where the phagosome merges with the lysosome, leading to the degradation of its contents [3,4]. Lysosomal pathways play a crucial role in breaking down long-lived proteins, insoluble protein aggregates, entire organelles, macromolecular compounds, and intracellular microbes (e.g., certain bacteria). This degradation can occur through endocytosis, phagocytosis, or autophagy pathways [5,6]. The UPS consists of proteasome that is a protease, ubiquitin ligases, and deubiquitinating enzymes (DUBs) [2]. Proteins condemned to decay are labeled with a covalently attached polyubiquitin chain and delivered to the proteasome for degradation. Ubiquitination is catalyzed by specific enzymes called E1 (activating enzyme), E2 (conjugating enzyme), and E3 (ubiquitin ligase) [3]. Monoubiquitinated proteins are degraded within lysosomes whilst polyubiquitinated ones are degraded in the UPS [4,5]. It was demonstrated that at least four ubiquitin molecules are needed for the proteasome to recognize it as a target protein [3]. Ubiquitylation is reversible. The removal of the polyubiquitin chain is catalyzed by DUBs [6]. Proteins marked for degradation are redundant, misfolded, or otherwise damaged (Figure 1A). The 26S proteasome, in which protein degradation takes place, can be divided into two subcomplexes: the 19S regulatory particle (RP) and the 20S core particle (CP). The function of the RP is to recognize, unfold, deubiquitylate, and translocate substrates to the CP—the site of proteolysis [7]. The 26S proteasome is crucial for maintaining protein and amino acid homeostasis, serving as a proteome modulator by degrading regulatory proteins. It plays a vital role in controlling various cellular processes such as the cell cycle, DNA replication, transcription, signal transduction, and stress responses [8]. Therefore, interference with the UPS may disturb cellular metabolism and even induce cell death.

It was hypothesized that proteasome inhibition may be a promising therapeutic approach. The first molecule designed to interfere with UPS was bortezomib (BTZ). Next generation proteasome inhibitors are carfilzomib and ixazomib [9,10]. Although the three molecules have a different chemical structure, BTZ and ixazomib structurally resemble each other as they are modified boronic acid derivatives [11,12]. Carfilzomib is a synthetic tetrapeptide epoxyketone [13]. It should be noted that only ixazomib can be administered orally [14].

BTZ turned out to be a potent chemotherapeutic agent that selectively and reversibly binds to the chymotryptic site located in the 20S proteasome, and therefore inhibits the degradation of ubiquitin-tagged proteins [11]. The blockage of these molecular pathways hampers protein degradation, leading to an accumulation of misfolded or otherwise defective polypeptides. These series of events ultimately lead to growth inhibition and apoptosis [15,16] (Figure 1B). BTZ can be administered intravenously or subcutaneously with the same efficiency. It was demonstrated that subcutaneous administration is as effective as intravenous. However, it is associated with a limited number of complications and adverse effects [17]. Once the drug reaches the circulation, it is rapidly removed from the plasma and distributed to the cellular compartment. The half-life of BTZ elimination is relatively long and is estimated to exceed 10 h [15]. BTZ undergoes oxidative metabolism in the liver via the cytochrome P450 complex to inactive deborated metabolites [18,19] and is excreted both through the kidneys and bile [20]. Despite its potent anticancer activity, patients treated with BTZ are at risk of developing certain adverse reactions. Multiple 1/2 phase clinical trials have been conducted investigating the safety and feasibility of BTZ in various indications, with particular emphasis on multiple myeloma (MM). Among the most frequent adverse reactions were gastrointestinal symptoms, fatigue, thrombocytopenia, neutropenia, and peripheral neuropathy [21,22,23,24,25,26,27]. There were no reports of BTZ cumulative toxicity [28,29,30,31]. BTZ alone and in combination with other agents was tested in both hematologic malignancies [32,33,34] and solid tumors such as breast, lung, and ovarian cancer [35,36,37,38]. BTZ is currently approved for the treatment of MM and mantle cell lymphoma [39].

The main mechanism of action of BTZ, i.e., proteasome inhibition, is relatively well described. Less is known about the epigenetic alterations that BTZ causes when administered to the organism. Epigenetics involves modifications in gene expression that are self-directed and resist explanation solely through changes in the nucleotide sequence [40]. These modifications may arise from external environmental influences or exposure to various factors, including drugs [41]. There are four main epigenetic mechanisms: DNA methylation, histone modification, chromatin remodeling, and non-coding RNA (ncRNA) [42]. The aim of this review is to provide greater insight into the epigenetic alterations induced by proteasome inhibitors, with particular emphasis on BTZ and its implications in the mechanism of action, the pathophysiology of adverse events, the development of resistance, and clinical implications. We decided to focus on BTZ as we have already investigated BTZ-resistance mechanisms and the role of epigenetic mechanisms in dodging BTZ-induced cytotoxicity [43,44,45,46]. Furthermore, it remains the most widely utilized proteasome inhibitor globally. Hence, we hypothesized that the vast majority of MM patients worldwide have been and will continue to be exposed to BTZ in the foreseeable future. Consequently, a more profound comprehension of the mechanisms of action of BTZ and the development of BTZ-resistance is of paramount importance. Such understanding may enhance patients’ outcomes, improve prognosis, and contribute to optimizing BTZ-based treatment regimens.

## 2. Epigenetic Alterations as an Important Part of BTZ’s Mechanism of Action

BTZ’s major mechanism of action, i.e., the inhibition of the 20S proteasome, is very well established and thoroughly described in the existing literature. However, less is known about its collateral mechanisms that also contribute to BTZ-related cell death. Since BTZ is an external stimulus for cells, it can be hypothesized that its presence, in addition to intracellular protein accumulation, has the proclivity to alter the methylome or promote other epigenetic alterations. Liu et al. showed that proteasome inhibition leads to protein aggregation, particularly affecting the Sp1 protein. Further analysis revealed that BTZ influences Sp1—the zinc finger protein—that transactivates the *DNMT1* gene (DNA methyltransferase) and is functionally regulated by its abundance. By inducing intracellular protein aggregation, BTZ reduces the levels of Sp1, disrupts its physical interaction with the NF-κB transcription factor, and consequently prevents the binding of the Sp1/NF-κB complex to the *DNMT1* gene promoter. The abrogation of the Sp1/NF-κB complex by BTZ causes the transcriptional repression of the *DNMT1* gene and the downregulation of the DNMT1 protein, leading to global DNA hypomethylation [47]. In a study conducted on mantle cell lymphoma (MCL) cell lines and in a mouse xenograft model, Leshchenko et al. demonstrated in a genome-wide analysis that BTZ administration causes a global loss of methylation, including the *Noxa* gene (a pro-apoptotic member of the Bcl-2 family) [48]. The *Noxa* gene is essential for BTZ cytotoxicity and BTZ-induced apoptosis, as *Noxa* depletion has been shown to abrogate BTZ efficacy [48,49].

Kikuchi et al. conducted an interesting study in which they demonstrated that histone deacetylases (HDACs) are critical targets of BTZ. Their results depicted that BTZ appeared to induce cytotoxicity in MM cells by suppressing HDACs. Furthermore, this phenomenon was accompanied by histone hyperacetylation, both occurring in a dose- and time-dependent manner. The most prominent effect was evident in the suppression of HDAC1. Conversely, the overexpression of HDAC1 rescued MM cells from BTZ-induced apoptosis [50]. Histone ubiquitylation is a potent epigenetic mechanism regulating gene expression and DNA damage repair [51]. BTZ has been shown to deplete histone H2B ubiquitination, triggering various downstream mechanisms that contribute to its cytotoxic activity [52,53].

## 3. Epigenetic Aspects of the Resistance to BTZ

Exposure to anticancer agents exerts tremendous environmental pressure on malignant cells and, over the course of time, selects for drug-resistant clones. Due to the fact that resistance develops as a consequence of an external trigger, such as anticancer treatment, it can be hypothesized that epigenetic alterations contribute, at least partially, to the development of drug resistance. Recent evidence suggests that this assumption also applies to BTZ. The proteasome serves as the molecular target for BTZ. Consequently, the notion that its downregulation could mitigate BTZ’s anti-tumor potential appears reasonable. This hypothesis was adopted by Tsvetkow and colleagues. They investigated the correlation between proteasome expression and sensitivity to BTZ, demonstrating that proteasome suppression, particularly the 19S subunit, was associated with BTZ resistance. Furthermore, the analysis of the underlying mechanisms revealed, among other factors, that the downregulation of *PSMD5* (the gene encoding one of the 19S subunits) due to promoter hypermethylation conferred resistance to BTZ [54]. In a neuroblastoma cell line, we demonstrated that exposure to BTZ elicited alterations in the methylome of cells that survived the treatment, i.e., cells exhibiting a resistant phenotype. The analysis of methylation profiles revealed that BTZ induced genome-wide changes in the methylome of those cells in comparison to both untreated and lenalidomide-treated controls. The alternations were not limited to CpG islands; in fact, the vast majority, approximately 90%, did not involve CpG islands. Most of the observed changes in methylation involved the loss of methylation. It is intriguing, however, that when the analysis is restricted to only significant changes in methylation, most of the observed alternations in the dataset appear to involve a loss or gain of methylation at around 50%. This may suggest that only one allele was affected. Overall, the results indicate that methylation changes may play a major role in the development of BTZ resistance [43].

In another in vitro study, Hu at al. investigated the relationship between the expression of CD9, a membrane molecule whose downregulation plays a role in cancer development and progression, and BTZ sensitivity, with particular emphasis on the epigenetic aspects of the underlying mechanisms. They demonstrated that BTZ-resistant MM cells exhibited significantly lower CD9 expression compared to cells with a sensitive phenotype. Since the CD9 promoter region includes a CpG island, a further analysis of the methylation profile was performed. The authors demonstrated an increase in the level of methylation in the promoter region of U266 and NCI-H929 MM cells with silenced CD9 expression. Moreover, CD9 expression was significantly induced after treatment with 5-Aza-2-deoxycytidine (AZA), a methylation inhibitor. In the aftermath, MM cells regained sensitivity to BTZ [55]. The deleted in colorectal cancer gene was shown to be involved in the carcinogenesis of various neoplasms. Rodrigues-Junior and colleagues investigated the role of DCC in myelomagenesis. They conducted an in vitro study on three different MM cell lines. The results showed that the hypermethylation of the promoter was associated with a better response to BTZ compared to SKO007 and U266, which were characterized by a low degree of *DCC* methylation and, consequently, its high expression. They not only demonstrated the role of *DCC* in the pathophysiology of MM, but also provided further evidence for the role of epigenetic changes in the sensitivity and resistance to proteasome inhibitors [56].

## 4. Targeting Epigenetic Mechanisms Restores Sensitivity to BTZ and Represents a Promising Therapeutic Strategy

### 4.1. Methylation Inhibitors Act Synergically with BTZ and Restore Sensitivity to This Compound

The aforementioned studies have shown that BTZ has the potential to induce epigenetic changes. Alterations in the methylome contribute to BTZ-related cytotoxicity on one hand and are an important aspect of BTZ’s mechanism of action. On the other hand, they have been demonstrated to confer BTZ resistance. Therefore, exploring the combination of BTZ with agents that modulate epigenetic changes may be an intriguing research area with potential clinical implications. Indeed, such combinations have been studied in vitro and in vivo. It may be challenging to compose an optimal treatment regimen due to the complexity of interactions and various treatment escape mechanisms. In order to facilitate the design of an effective chemotherapy regimen, Rashid et al. developed the quadratic phenotypic optimization platform (QPOP), a tool to aid in the design of optimal drug combinations in MM. The application of QPOP to BTZ-resistant MM cell lines identified drug combinations that collectively optimized treatment efficacy. The QPOP project determined a drug combination countering DNA methylation, with decitabine (DAC) being one of the selected agents. The results were validated in vivo using a mouse model. DAC and mitomycin C were demonstrated as a potent drug combination in the treatment of BTZ-resistant MM [57].

We demonstrated that AZA acts synergically with BTZ, and a combination of AZA and BTZ exhibited cytotoxic effects against BTZ-resistant U266 MM cells [45]. A similar effect regarding AZA and BTZ was demonstrated by Li and colleagues [58]. Qi et al. provided further evidence supporting the effectiveness of the combination of BTZ and a methylation inhibitor. They conducted an in vitro experiment on bladder cancer cell lines, demonstrating that BTZ-resistant cells had a low expression of *HSPA1A* which was associated with the revealed hypermethylation of the *HSPA1A* promoter. This gene, next to *HSPA1B*, encodes heat shock protein 72 (HSP72), or more precisely, various isoforms of HSP72. The combination of AZA and BTZ restored BTZ sensitivity in previously resistant bladder cancer cells [59]. Similarly, BTZ and another DNMT inhibitor, decitabine (DAC), have been shown to act synergically against BTZ-resistant clones both in vitro and in a xenograft animal model [48]. Another study investigated the effects of DAC alone, BTZ alone, and DAC combined with BTZ on MM cell viability. The results showed that DAC alone inhibited the growth of MM cells, while the combination of BTZ and DAC showed a synergistic effect. The primary molecular anti-MM effects of DAC and BTZ were shown to be induced by the modulation of the Wnt/β-catenin pathway. These observations were first established in MM cell lines and subsequently confirmed in a mouse xenograft model [60]. RRx-001 (1-bromoacetyl-3,3-dinitroazetidine) is an innovative epigenetic modulator that operates differently from classic epigenetic drugs, such as AZA or DAC. It allosterically modifies hemoglobin and, under hypoxic conditions, catalyzes the conversion of nitrite to bioavailable nitric oxide (NO), which accumulates in poorly oxygenated tumors. NO further generates free radicals and causes oxidative stress. Thus, RRx-001 exerts stress on malignant cells, leading to the inhibition of DNMT and global hypermethylation, along with the restoration of tumor suppressor gene function. RRx-001 has been shown to inhibit growth, induce apoptosis, and overcome BTZ resistance in MM cells [61]. The effectiveness of methylation inhibition was also confirmed in other studies [62].

Although methylation is typically associated with well-known regulatory regions of a gene, such as the promoter, it also applies to other areas of the nucleotide sequence. For instance, Xu and colleagues investigated the role of a relatively novel epigenetic regulatory mechanism, N^6^-methyladenosine (m6A), which involves methylation at the N6 position of adenosine. They showed that fat mass and the obesity-associated protein (FTO), m6A demethylator, are upregulated in MM, particularly with extramedullary location, and FTO inhibition was toxic towards MM cells. Moreover, cytotoxicity was significantly enhanced when the FTO inhibitor was administered together with BTZ, so the combination of a proteasome inhibitor and drug interfering with the epigenetic mechanism exerted synergistic anti-MM effects [63]. Similarly, Jia et al. showed that WTAP, a key component of the m6A methyltransferase complex, was methylated by Protein Arginine Methyltransferase 1 (PRMT1), and the combination of a PRMT1 inhibitor and BTZ synergistically inhibited MM progression [64].

### 4.2. Therapeutic Interference with Histone Modifications

Histone modifications are another epigenetic mechanism that has been studied in BTZ-treated MM. Sun and colleagues investigated Nexturastat A (NexA), a selective histone deacetylase 6 (HDAC6) inhibitor. In vitro NexA inhibited the growth and induced the apoptosis of RPMI-8226 and U266 MM cells, including cells that were resistant to BTZ. Those results were further confirmed in a mouse xenograft model [65]. Cytogenetic abnormalities are a hallmark of MM and serve to stratify patient risk and thus predict prognosis and clinical outcomes to some extent [66,67]. Translocation t(4;14) is relatively common and is associated with a poor prognosis even in the era of novel anti-MM agents, including monoclonal antibodies [68]. Jiang and colleagues demonstrated that Aurora kinase A phosphorylates NSD2 at the S56 residue to enhance NSD2 methyltransferase activity, conferring resistance to BTZ. A selective Aurora kinase A inhibitor (MLN8237) presented a prominent synergistic effect with BTZ on MM cells with t(4;14). Interestingly, such observations were limited to MM cells with t(4;14) translocation. The methylation of Aurora A and the phosphorylation of histone methyltransferase NSD2 bilaterally form a positive regulatory loop that promotes BTZ resistance in MM cells. It should be emphasized that these observations were further confirmed in an in vivo model [69]. Similarly, Liu and co-workers demonstrated the overexpression of NSD2 in BTZ-resistant MM cells and in cells obtained from patients with the t(4;14) translocation. It was found that there was a significant upregulation of NSD2 resulting in an increase in steroid receptor coactivator-3 (SRC-3). Elevated levels of both SRC-3 and NSD2 were confirmed in BTZ-resistant MM cells, irrespective of cytogenetic background. Importantly, the SRC-3 inhibitor, SI-2, restored BTZ sensitivity in vitro and in a xenograft model. Notably, SI-2 promoted bone-lesion recovery in mice. The study concluded that the histone methyltransferase NSD2 stabilized SRC-3 protein levels, and pharmacological interference with SRC-3 abrogated this interaction, resynthesizing MM cells to BTZ in both in vitro and in vivo models [70].

The synergic effect of histone deacetylase inhibitors and BTZ is not limited to MM. For instance, Bollmann and colleagues conducted an important and interesting study in which they demonstrated that a novel selective histone deacetylase inhibitor, named YAK540, and BTZ enhance each other’s cytotoxic effects on leukemia cell lines. These effects were exerted through the increased expression of pro-apoptotic genes, increased p21 expression, and caspase 3/7-mediated apoptosis [71]. Chidamide is a novel benzamide inhibitor of HDAC. Xu et al. demonstrated that chidamide repressed autophagy, which, similarly to UPS, participates in intracellular protein degradation, and synergically with BTZ inhibits MM cell growth. They provided compelling evidence that excessive proteotoxic stress could, at least in part, explain the underlying molecular effects of chidamide in combination with BTZ against MM [72]. The bone marrow microenvironment and its interaction with MM cells plays a vital role in mediating acquired resistance to BTZ, for instance, through Jagged1-induced Notch activation in myeloma cells (Jagged1 is widely expressed in various types of cells within the bone marrow MM niche) [73]. In the context of overcoming bone marrow microenvironment-dependent BTZ resistance through epigenetic mechanisms, Sripayap et al. showed that the HDAC inhibitor Romidepsin can counteract cell adhesion-mediated drug resistance [74].

## 5. The Role of Non-Coding RNAs

In addition to DNA methylation, non-coding RNAs such as mi-RNA or long non-coding RNA (lncRNA) serve as potent epigenetic regulators of gene expression and protein synthesis. Related processes were also investigated regarding the BTZ’s mechanism of action and the development of resistance to this compound. Non-coding RNAs mediate BTZ-induced cytotoxicity and while this area is not entirely elucidated, several underlying mechanisms have been identified. For example, following BTZ exposure, the transcription factor CEBPD is activated, which triggers the transcriptional activation of miR-744, miR-3154, and miR-3162. These miRNAs form a complex with Ago2 and move into the nucleus to target their complementary DNA sequence-binding sites on the promoter regions of four important genes—*CEBPD*, *PRKDC*, *MCM4*, and *UBE2V2*. The initiator miRNAs/Ago2 complex interacts with YY1 and recruits the epigenetic regulators, the PcG complex/DNMTs, to silence the four gene loci, including CEBPD itself. The inactivation of these potent oncogenes, *PRKDC*, *MCM4*, and *UBE2V2*, causes leukemic cell death through epigenetic silencing mediated by CEBPD-responsive miRNA [75]. Another non-coding RNA being investigated is circ_0007841. First, its overexpression was established in MM compared to healthy controls. In addition, patients with a low expression of circ_0007841 had a higher survival rate compared to those with high circ_0007841 levels. Subsequently, circ_0007841 depletion was shown to impede MM cell proliferation and promote apoptosis. The knock out of circ_0007841 reduced the BTZ resistance of MM cells in vitro and MM growth in a mouse xenograft model. Hence, it can be hypothesized that the overexpression of circ_0007841 confers, at least to some extent, resistance to BTZ [76].

Another non-coding RNA being investigated in MM is miR-29b. It has been shown to inhibit DNMT and thus reduce global DNA methylation in MM cells [77]. Moreover, miR-29b was demonstrated to impede MM cell migration [78]. Of particular interest regarding MM treatment, miR-29b was upregulated by BTZ and was involved in BTZ-related cytotoxicity [79]. Therefore, molecules mimicking miR-29b or its analogues may represent a potential novel therapeutic approach in the treatment of MM. Pan et al. showed that lncRNA H19 mediates BTZ resistance in MM cell lines and promotes tumor growth in vivo. First, they showed that lncRNA is highly expressed in the serum of BTZ-resistant patients [80]. Subsequently, they conducted another study to elucidate the underlying mechanisms. They found that BTZ resistance is mediated by lncRNA H19 through the overexpression of MLC-1, an anti-apoptotic protein belonging to the Bcl-2 family. To be more specific, lncRNA H19 interacts with miR-29b-3p, a physiological regulator of MLC-1 expression. The interaction between H19 and miR-29b-3p upregulates MLC-1, enhancing its anti-apoptotic properties and thus promoting BTZ resistance [81]. The role of miR-29b in pathogenesis was also highlighted by Fu et al. The authors demonstrated that lncRNA myocardial infarction-associated transcripts (MIATs) were highly expressed in patients with MM and interacted with miR-29b to negatively regulate its expression. Moreover, experimental evidence demonstrated that MIATs increased BTZ resistance in MM cells by targeting miR-29b [82].

Qin et al. showed that miR-137 is epigenetically silenced by promoter methylation in MM, and the entire process is reversible after using AZA. What is particularly interesting is that the overexpression of miR-137 sensitized cells to BTZ (in vitro and in a murine xenograft model) and overcame chromosomal instability [83]. Wu et al. demonstrated miR145-3p to be downregulated in MM patients compared to healthy donors. Moreover, its expression was correlated with markers of disease progression. The researchers further demonstrated that induced miR145-3p expression inhibited cell proliferation and promoted apoptosis in MM cells by inducing autophagy. The underlying mechanism was associated with HDAC4 inhibition. Importantly, the upregulation of miR-145-3p enhanced the anti-MM activity of BTZ. The latter has also been demonstrated in a mouse xenograft model [84]. Consistent with the results obtained by Nian and colleagues, lncRNA ANGPTL1-3 expression was correlated with MM International Staging System (ISS) and OS. Furthermore, they demonstrated that this molecule mediates resistance to BTZ via interaction with miR-30a-3p and the transcription factor c-Maf [85]. Other non-coding RNAs that have been proven to mediate BTZ resistance are circ-CCT3 by modulating the miR-223-3p/BRD4 axis [86], miR-214-3p, miR-5100 [87], and several others [88,89,90,91,92,93].

Moreover, Malek et al. identified an entire panel of deregulated lncRNAs mediating acquired resistance to three different clinically relevant proteasome inhibitors, i.e., BTZ, carfilzomib, and ixazomib in MM [94]. Additionally, the knockdown of lncRNA PCAT-1 inhibits myeloma cell growth and enhances sensitivity to BTZ [95]. Conversely, some non-coding RNAs such as miR-197-3p [96], miR-631 [97], miR-497 [98], miR-155 [99], and miR-200c [100] have been shown to reduce BTZ resistance. Another particular aspect is the synergistic interference with proteasome function and autophagy, enhancing BTZ’s anti-MM properties. For example, non-coding RNAs, including lncRNA MEG3, have demonstrated the ability to influence autophagy, thus acting synergistically with BTZ to promote sensitivity in MM [101]. A similar observation regarding the inhibition of autophagy by chidamide was mentioned above, further suggesting the importance of this finding [72]. In addition, Jagannathan et al. showed that concomitant interference with proteasome and autophagosome through miR-29b replacement enhances the anti-MM effect of BTZ [102].

**Figure 2:**
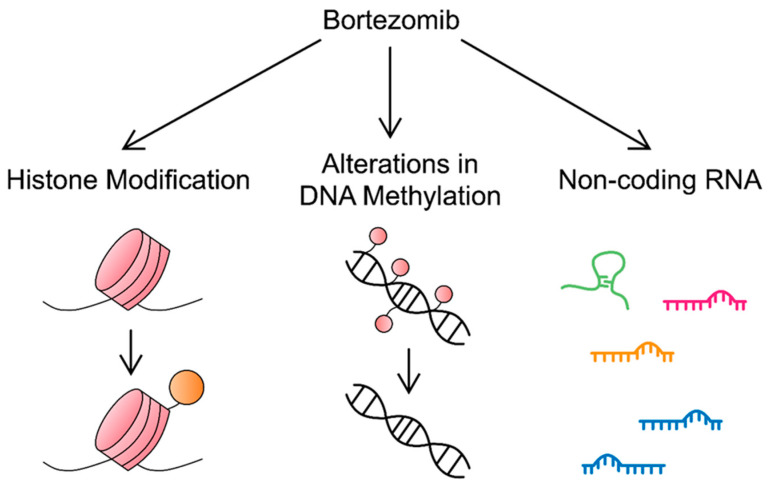
Epigenetic alterations elicited by BTZ. In addition to proteasome inhibition, BTZ exerts its cytotoxic effects through histone modifications, alterations in DNA methylation (mainly, loss of methylation), and non-coding RNs. Similar epigenetic alterations contribute to the development of resistance to this compound.

All of the above-mentioned molecules and mechanisms involved in mediating BTZ resistance or enhancing its cytotoxic effects against MM (Figure 2) are highly significant, given their possible clinical implications and targetability. This could potentially translate into therapeutic strategies in the future.

## 6. Peripheral Neuropathy

In addition to contributing to the development of BTZ resistance, epigenetic alterations may play a role in the pathogenesis of its adverse reactions. Łuczkowska et al. investigated the pathophysiology of BTZ-induced peripheral neuropathy. As neuropathic symptoms may partially resolve upon discontinuation of BTZ, the researchers hypothesized that epigenetic changes may, at least in part, mediate the development of peripheral neuropathy. First, they demonstrated that BTZ induces global hypomethylation in neuronal cells. Interestingly, their results revealed an increase in methylation at a particular subset of CpG sites. Nevertheless, they were present outside the CGI and gene regulatory regions. Further GSEA analysis revealed that these changes appeared to affect genes involved in morphogenesis, neurogenesis, and neurotransmission. Moreover, the identified methylation changes are significantly enriched within the binding sites of transcription factors, including EBF, PAX, DLX, LHX, and HNF family members. The study concluded that alterations in the methylome are likely to be involved in BTZ-mediated neurotoxicity [44].

In addition to alterations in the methylome, the researchers investigated other epigenetic alterations putatively being involved in BTZ-induced peripheral neuropathy. The obtained results showed a decrease in global histone H3 acetylation. Furthermore, miR-6810-5p has been shown to interfere with the *MSN*, *FOXM1*, *TSPAN9*, and *SLC1A5* genes, which are involved in neuroprotective processes, neuronal differentiation, and signal transduction [103]. Zheng and colleagues demonstrated that the activation of GATA-binding protein 3 (GATA3) mediated the epigenetic upregulation of CCL21 in dorsal horn neurons, which contributed to BTZ-induced neuropathic pain. More precisely, BTZ induced histone hyperacetylation in the *CCL21* gene promoter via GATA3 signaling [104]. The role of histone hyperacetylation in BTZ-induced allodynia was also observed by Chen and colleagues [105] and Liu and co-workers [106]. Similarly, the overexpression of the histone demethylase KDM6A has been shown to participate in BTZ-induced neuropathic pain [107]. Parallel observations regarding the contribution of epigenetic alterations in the pathophysiology of peripheral neuropathy development were made by Liu and colleagues [108]. Furthermore, the analysis of patients with BTZ-induced peripheral neuropathy revealed increased plasma levels of various miRNAs. miR-22-3p, miR-23a-3p, and miR-24-3p have been identified as potential biomarkers of peripheral neuropathy [109].

BTZ-induced peripheral neuropathy has a multifactorial pathogenesis. Several mechanisms were postulated, including inflammatory background [110]. Nonetheless, the above studies depicted a complex interplay between various epigenetic and genetic mechanisms. The hypothesis of the involvement of epigenetic changes in the development of BTZ-induced peripheral neuropathy seems to be convincingly confirmed, yet it needs to be further investigated. It should be emphasized, however, that the development of this complication is also, most likely to a large extent, influenced by other factors.

## 7. Clinical Implications

In addition to in vitro studies, the significance of epigenetic changes in clinical settings has been investigated. For instance, De Larrea et al. researched the clinical implications of alterations in the methylome in MM. They analyzed the methylation profile of seventy-five MM patients treated with BTZ-based regimens. Bone marrow samples were obtained at the time of relapse. Global methylation was determined using ELISA and the CpG island DNA methylation profile of 30 genes using a PCR system. The results showed that MM patients with more than 3.95% of total DNA methylated achieved better overall survival (OS) than patients with more unmethylated DNA (median 30 versus 15 months, *p* = 0.004). Then, the methylation level of individual genes was analyzed. The results showed that a methylation status lower than 3.97% in CXCR4 was correlated with longer progression-free survival (PFS) after BTZ treatment. Subsequently, cluster analysis of all thirty genes was conducted. It was demonstrated that *NFKB1* was the only gene associated with a differential profile to BTZ, showing that responders to the treatment exhibited a lower methylation status (*p* = 0.029). A low percentage of methylation (less than 1.07%) in this gene was also associated with longer overall survival (OS) after BTZ exposure. The study concluded that the combination of relatively low levels of global genome methylation (<3.95%) and higher levels of *NFKB1* methylation (≥1.07%) identified a specific subset of patients with extremely short OS [111].

Szudy-Szczyrek et al. investigated the predictive and prognostic value of miR-8074 expression in MM patients. They analyzed 105 patients with newly diagnosed MM treated with thalidomide (THD) (*n* = 27), BTZ (*n* = 41), and both BTZ and THD (*n* = 37). The obtained results showed that a high expression of miR-8074 was associated with a worse clinical outcome, more precisely with a higher risk of death (HR = 4.12, 95% CI: 2.20–7.70; *p* = 0.0009) and with a significant reduction of PFS. This renders miR-8074 a useful tool for predicting the prognosis for MM patients [112]. Another non-coding RNA associated with clinical outcomes is miR-137. It was demonstrated that the expression of this molecule is negatively correlated with PFS and OS [83].

As mentioned in the sections above, there is ample evidence that epigenetic alterations contribute to the development of BTZ resistance. Furthermore, targeting epigenetic mechanisms either resynthesized cells to BTZ or exhibited a synergistic effect with the drug. In addition, changes in methylome influenced prognosis.

Therefore, it seems reasonable to conduct a clinical trial examining the combination of BTZ and a molecule influencing epigenetic mechanisms. Panobinostat, a first-in-class pandeacetylase inhibitor (DACi), is a molecule interfering with epigenetic mechanisms. More specifically, it prevents deacetylation, a process involved in epigenetic regulation [113]. It was demonstrated that panobinostat is a viable therapeutic option for MM patients. Results from PANORAMA1, a multicenter, randomized, double-blind, placebo-controlled, phase 3 trial, demonstrated that patients treated with the combination of panobinostat, BTZ, and dexamethasone (PAN-BTZ-Dex) benefited in terms of OS compared to placebo (BTZ and dexamethasone alone) [114]. Therefore, the inhibition of a proteasome and interference with epigenetic alterations exert synergistic anti-MM effects. A subgroup analysis included patients who had received a prior immunomodulatory drug (IMiD) or BTZ plus IMiD or ≥2 prior regimens including BTZ and IMiD. This analysis demonstrated a clear benefit in terms of PFS with PAN-BTZ-Dex among patients who had received ≥2 prior regimens containing BTZ and IMiD, a subgroup of patients with limited therapeutic options and a worse prognosis [115]. Studies conducted in a clinical setting are summarized in Table 1.

## 8. Conclusions

The implementation of BTZ, a first-in-class proteasome inhibitor, was a gamechanger in the treatment of MM. It not only improved the clinical outcomes of MM patients, but also laid the foundation for the further development of next-generation molecules, i.e., carfilzomib and ixazomib. In addition to proteasome inhibition, the effects of BTZ are closely related to epigenetic changes. First, BTZ cytotoxic effects are mediated, among others, through a global decrease in methylation in target malignant cells. Therefore, hypomethylation is a vital aspect of BTZ’s mechanism of action. Furthermore, the development of BTZ resistance is also associated with changes in the methylome. In addition, drugs interfering with epigenetic mechanisms, such as AZA or DAC, have been shown to be effective in two different ways. In the first place, it should be mentioned that they have been demonstrated to restore the BTZ sensitivity of BTZ-resistant malignant clones. Moreover, in combination with BTZ, they have a synergistic effect on cells previously not exposed to the proteasome inhibitor. Finally, epigenetic alterations contribute to the development of BTZ adverse effects, such as drug-induced peripheral neuropathy. In addition to various changes in global methylation status, the role of non-coding RNA and histone modifications, particularly in patients with certain cytogenetic abnormalities, is still a subject of ongoing research.

It should be noted that majority of studies were conducted in vitro. Therefore, it is of a paramount importance to further explore this area in a clinical setting. This approach would lead to clinically relevant results that could improve patient outcomes.

## 9. Future Perspectives

BTZ induces genome-wide methylation changes, which, on one hand, are part of its mechanism of action. On the other hand, they mediate the development of BTZ resistance. The reversibility of methylation changes renders them an interesting research area and a potential therapeutic strategy. Methylation inhibitors have already been shown to act synergistically with BTZ and counteract BTZ resistance when administered to resistant clones. However, there are aspects of epigenetic alterations that need to be further explored. There are novel molecules with a dual mechanism of action, targeting two different epigenetic modifications. They inhibit G9a and DNMT simultaneously. Their efficacy has been confirmed in the treatment of solid tumors such as cholangiocarcinoma and hepatocellular carcinoma [113,114] and several hematologic malignancies, be it acute myeloid leukemia (AML), acute lymphoblastic leukemia (ALL), or diffuse large B-cell lymphoma (DLBCL), both in cell lines and in mouse xenograft models [115]. A similar molecule that, in addition to inhibiting G9a and DNMT, also interfered with histone deacetylases was tested in MM. The results revealed that the anti-MM effects were achieved through histone-3 acetylation, DNA hypomethylation, and decreased histone-3 methylation at lysine-9. Efficacy was confirmed first in MM cell lines and subsequently in a mouse xenograft model [116]. Despite promising results, these molecules have not been tested in combination with other drugs. The combination of epigenetic agents with BTZ may be investigated due to the fact that both BTZ cytotoxicity and the development of resistance are largely dependent on epigenetic changes, which renders this an interesting research direction.

## Figures and Tables

**Table 1:** Clinical implications of epigenetic alterations in MM treated with BTZ-based regimens.

Qin et al. [83]	miR-137 increases sensitivity to BTZ whilst the low expression of miR-137 is associated with shorter OS and PFS.
De Larrea et al. [111]	The hypomethylation of *NFKB1* is associated with good response to BTZ and better OS. More than 3.95% of total methylated DNA correlates with better OS.
Szudy-Szczyrek et al. [112]	The high expression of miR-8074 is associated with a higher risk of death and shorter PFS in MM exposed to BTZ and THD.
San-Miguel et al. [113,114]; Richardson et al. [115]	The combination of Panobinostat (epigenetic drug) with BTZ and dexamethasone is an effective treatment regimen.

## Data Availability

The data presented in this study are available in this article.
